# Clinical characteristics, outcomes, and subtype diversity in hospitalized human rhinovirus (HRV) patients

**DOI:** 10.1371/journal.pone.0335739

**Published:** 2025-11-05

**Authors:** Menucha Jurkowicz, Michael Solomovich, Eugene Leibovitz, Nathan Keller, Dafna Yahav, Galia Barkai, Nofar Atari, Ilana S. Fratty, Hodaya Cohen, Ana Belkin, Yaniv Lustig, Michal Stein, Michal Mandelboim

**Affiliations:** 1 Department of Epidemiology and Preventative Medicine, Faculty of Medical and Health Sciences, School of Public Health, Tel Aviv University, Tel Aviv, Israel; 2 Central Virology Laboratory, Ministry of Health, Chaim Sheba Medical Center, Ramat Gan, Israel; 3 Pediatric Infectious Disease Unit, The Edmond and Lily Safra Children’s Hospital, Chaim Sheba Medical Center, Ramat Gan, Israel; 4 Faculty of Medical and Health Sciences, School of Medicine, Tel Aviv University, Tel Aviv, Israel; 5 Faculty of Health Sciences, Ben-Gurion University of the Negev, Beer-Sheva, Israel; 6 Ariel University, Ariel, Israel; 7 Infectious Disease Unit, Chaim Sheba Medical Center, Ramat Gan, Israel; 8 The Israel Center for Disease Control, Ministry of Health, Tel Hashomer, Israel; Weill Cornell Medicine - Qatar, QATAR

## Abstract

**Background:**

Human rhinovirus (HRV) is a major cause of respiratory illness, however data on clinical presentation, outcomes across age-groups and associations with HRV subtypes are limited.

**Methods:**

Clinical characteristics and outcomes of hospitalized HRV-positive patients with cycle threshold (Ct)≤32 were collected retrospectively and analyzed in relation to age-groups and subtypes.

**Results:**

Among 738 patients, the age distribution was: 0–1 (148,20.1%), 1–3 (94,12.7%), 3–5 (44,5.9%), 5–18 (76,10.3%), 18–40 (51,6.9%), 40–65 (95,12.9%) and ≥65 (230,31.2%). Younger children more frequently presented with bronchiolitis and asthma exacerbation, while older adults experienced higher rates of pneumonia and chronic obstructive pulmonary disease (COPD) exacerbation. ICU admissions and mechanical ventilation were more common in younger children, whereas oxygen support was predominant in older adults. Of 119 sequenced samples, HRV-A was the predominant species (69%), followed by HRV-C (28.5%), with both exhibiting considerable genetic subtype diversity. Lower respiratory tract infection (LRTI) associated with HRV-C was found only in adults while severe and critical outcomes with HRV-A and HRV-C occurred in both children and adults. When compared with human metapneumovirus (hMPV), a known pathogenic respiratory virus, no differences in severe outcomes were noted, however, HRV patients aged ≥65 had a higher proportion of critical outcomes.

**Conclusions:**

HRV infection is associated with significant morbidity across age-groups, with distinct clinical presentation and outcomes. ICU admissions were more frequent in children, while older adults required oxygen support. The genetic diversity and age-related differences in HRV subtypes underscore its clinical impact in both pediatric and adult populations.

## Introduction

Human rhinovirus (HRV) is a positive-sense, single-stranded, non-enveloped, RNA virus. It belongs to the *Enterovirus* genus, *Picornaviridae* family, and is subdivided into three species (A, B, and C), with over 150 known subtypes [1]. HRV is a main cause of acute upper respiratory illnesses including the common cold, otitis media, and sinusitis [2], however an association with severe morbidity is less established. In some patients, especially those with immunosuppression, or with comorbidities such as asthma or chronic obstructive pulmonary disease (COPD), the elderly or young children, HRV can cause severe illness, including lower respiratory tract infections such as pneumonia, bronchiolitis, exacerbation of existing asthma or COPD, and may even result in intensive care unit (ICU) admission or mechanical ventilation [2–4].

In immunocompetent patients, HRV shedding usually last for 10–14 days and is not always correlated with respiratory symptoms [5]. Prolonged HRV shedding for more than 5 weeks has been observed in young children following a symptomatic episode while chronic viral carriage over 4–12 months has been reported in immunocompromised patients [5].

The presence of respiratory viral agents is associated, in general, with respiratory symptoms, but the proof of a direct relationship between the detected virus and diagnosed symptoms is complex. The conventional detection methods (virus culture and virus serology) have shown relatively low mean detection rates for viruses in respiratory samples of asymptomatic subjects, while the detection rates (by conventional methods) were reported from 16% to 53% for various respiratory illnesses [6]. The use of polymerase chain reaction (PCR) techniques has increased virus detection rates not only in symptomatic but also in asymptomatic subjects and has made the interpretation of positive test results controversial. In children <4 years of age, rates of asymptomatic HRV infection reached 12–32% [2,6–8] and were not significantly different than those observed in children hospitalized with lower respiratory tract illnesses. In adults, the rates of asymptomatic HRV carriage are less well studied, but still much lower (0–2%) than those reported in children [9,10].

hMPV is a respiratory virus that is recognized as a significant cause of acute respiratory illness (ARI), especially in young children and older adults. hMPV is associated with a spectrum of clinical symptoms ranging from mild upper respiratory tract infections to more severe LRTIs [11]. Common symptoms of hMPV infection include fever, cough, rhinorrhea, wheezing, dyspnea, otitis media, bronchiolitis, and pneumonia [11], with severe cases sometimes requiring hospitalization and mechanical ventilation. In our study, we compared clinical outcomes of hMPV with those of HRV in order to better understand the clinical severity of HRV infection.

Limited studies have focused on the clinical presentation and outcomes of HRV infection across various age groups, or their association with HRV subtypes. We conducted a detailed retrospective analysis of the epidemiological, clinical and virologic characteristics of HRV infections in a hospitalized patient population in Israel and evaluated the association between HRV subtype and clinical outcomes.

## Methods

### Epidemiological analysis

A single-center, retrospective cohort study was performed, including all hospitalized patients with laboratory confirmed HRV at Sheba Medical Center (a 2,000-bed, tertiary, university affiliated hospital in Israel) from November 2020 through July 2022. Demographic, clinical, laboratory, management and outcome data were retrieved using MDClone ADAMS platform, a self-service query tool that provides comprehensive patient-level data of wide-ranging variables in a defined time frame around an index event (mdclone.com). The study was approved by the hospital’s institutional review board, approval number 9666-22-SMC. The data collection process was conducted from June 4 to September 12, 2023. Electronic health records were accessed to obtain data on intensive care unit (ICU) patients, missing data, and for data verification purposes. All data was kept confidential and anonymized, in accordance with ethical and privacy standards, and no information that could directly identify patients was used or disclosed.

The case analysis was completed according to patient age (divided into six groups; 0–1, 1–3, 3–5, 5–18, 18–40, 40–65 and >65 years of age) and HRV species and subtype. Cycle threshold (Ct) values were used as a surrogate measure of viral load, as previously described [12,13]. Severe outcomes were defined as room air saturation levels ≤92%. Critical outcomes were defined as a composite of either ICU admission, mechanical ventilation or vasopressor support within 30 days.

Exclusion criteria consisted of non-hospitalized HRV-positive patients, patients with CT > 32, and patients with co-detection of other respiratory viruses (respiratory syncytial virus [RSV], influenza A & B, parainfluenza, adenovirus, SARS CoV-2, and human metapneumovirus [hMPV]).

A comparative cohort consisted of 855 hMPV patients, hospitalized between 2015–2021.

### Laboratory methods

#### Viral nucleic acids extraction and real time PCR assay.

Clinical samples were collected from hospitalized patients presenting with respiratory symptoms during the study period. Viral RNA/DNA was extracted using the STARMag Viral DNA/RNA 200C universal kit (Seegene Inc., South Korea). The presence of HRV was tested using the Allplex™ RV multiplex real-time RT-PCR kit (Seegene Inc., South Korea). The viruses detected by this kit are influenza A, influenza B, hMPV, RSV, parainfluenza [1–4], adenovirus, SARS-CoV-2 and HRV [14,15]. The primers used to identify the viruses are not disclosed by the manufacturer. Extraction and RT-PCR procedures were conducted in accordance with the manufacturers’ instructions.

#### Sequencing and genomic analysis.

Sequencing was performed using the Sanger method and analyzed using the Sequencher program (Gene Codes Corporation, United States). qRT-PCR was performed on positive HRV samples using the One-Step RT-PCR kit (Qiagen, Germany), separated on a 2% agarose gel, and visualized by agarose gel electrophoresis. The primers used for sequencing were previously described, and are listed in S1 Table [16]. PCR products were purified with the EPPiC Fast enzyme (A&A Biotechnology, Poland). The DNA templates were sequenced using the BigDye Terminator v1.1 kit on an ABI Prism 3100 automated sequencer (Applied Biosystems, United States). Phylogenetic analysis of HRV species was performed by Geneious software (Dotmatics, New Zealand) using the neighbor-joining method with 1000 bootstrap replicates.

### Statistical analysis

Univariate analysis was conducted to identify differences across age groups and HRV subtypes in demographics, comorbidities and clinical characteristics during hospitalization. Variables were reported as counts and percentages for categorical variables, and as either mean with standard deviation (SD) or median with interquartile range (IQR) for continuous variables, depending on the distribution of the data. Categorical values across age groups were assessed using the chi-square test, or Fisher’s exact test when expected cell counts were ≤5. Trends across age groups were assessed using the Cochran-Armitage trend test, and p-values were reported as Trend P. Parametric continuous variables across age groups were assessed using one-way ANOVA, while nonparametric continuous variables were assessed using the Kruskal-Wallis test. Virologic species characteristics associated with severe or critical outcomes were assessed using Fisher’s exact test, while subtypes stratified by clinical outcomes were summarized descriptively without hypothesis testing due to the sparseness of data. Ct values were analyzed across age groups using the Kruskal-Wallis test, and Spearman’s rank correlation was used to evaluate associations between Ct and severe and critical outcomes. When comparing outcomes of HRV with hMPV, proportions were compared by age group using the chi-square or Fisher’s exact tests; borderline and significant p-values were reported. For all analyses, two-sided p-values <0.05 were considered statistically significant. All statistical analysis was performed using R Statistical Software (version 2024.04.1 + 748; R Foundation for Statistical Computing, Vienna, Austria).

## Results

### Clinical characteristics of HRV patients

A total of 13,803 respiratory samples were collected during the study period, with the positivity rate per virus as follows: 3.3% influenza (n = 449), 3.9% RSV (n = 544), 2.3% hMPV (n = 316), 3.2% adenovirus (n = 441), 3.4% parainfluenza (n = 470), and 13.6% HRV (n = 1,873). Of the 1,873 HRV patients, 1,135 patients were excluded as they had a Ct > 32, were not hospitalized, or were co-infected with another respiratory virus, resulting in a final cohort of 738 patients. Of these, 148 (20.1%), 94 (12.7%), 44 (5.9%), 76 (10.3%), 51 (6.9%), 95 (12.9%) and 230 (31.2%) belonged to age groups 0–1, 1–3, 3–5, 5–18, 18–40, 40–65 and >65 years, respectively (**Table 1**). Comorbidities including chronic cardiovascular disease, chronic lung disease, neurological disease and chronic kidney disease were more common among patients aged ≥65 years (Cochrane Armitage trend p < 0.01 for all listed comorbidities). The majority of patients with immune deficiency were recorded among age groups 3–5 (25.0%), 5–18 (31.6%), 18–40 (27.5%) and 40–65 (25.3%).

**Table 1:** Comparison of demographics and comorbidities in various age groups among HRV hospitalized patients.

	0-1 years	1-3 years	3-5 years	5-18 years	18-40 years	40-65 years	≥65 years	P-value	Trend P^d^
	*n = 148*	*n = 94*	*n = 44*	*n = 76*	*n = 51*	*n = 95*	*n = 230*		
**Female (n, %)**	68 (45.9)	40 (42.6)	19 (43.2)	33 (43.4)	24 (47.1)	45 (47.4)	108 (47.0)	0.99	
**Basal metabolic index (BMI) ≥30 (n, %)**	0 (0.0)	0 (0.0)	0 (0.0)	5 (8.1)	9 (19.6)	27 (29.7)	45 (21.1)	**<0.01**	**<0.01**
**Current or past smoker (n, %)**	0 (0.0)	0 (0.0)	0 (0.0)	0 (0.0)	15 (29.4)	29 (30.5)	47 (20.4)	**<0.01**	**<0.01**
**Pregnant or postpartum (n, %)**	0 (0.0)	0 (0.0)	0 (0.0)	0 (0.0)	2 (3.9)	1 (1.1)	0 (0.0)	**<0.01**	
**Cardiovascular disease (n, %)**	2 (1.4)	2 (2.1)	1 (2.3)	4 (5.3)	2 (3.9)	10 (10.5)	109 (47.4)	**<0.01**	**<0.01**
**Neurological diseases (n, %)**	6 (4.1)	9 (9.6)	6 (13.6)	9 (11.8)	10 (19.6)	11 (11.6)	53 (23.0)	**<0.01**	**<0.01**
**Chronic lung disease (n, %)**	0 (0.0)	2 (2.1)	0 (0.0)	3 (3.9)	4 (7.8)	25 (26.3)	77 (33.5)	**<0.01**	**<0.01**
**Asthma (n, %)**	0 (0.0)	4 (4.3)	7 (15.9)	13 (17.1)	6 (11.8)	14 (14.7)	13 (5.7)	**<0.01**	
**Immune deficiency** ^a^ **(n, %)**	7 (4.7)	10 (10.6)	11 (25.0)	24 (31.6)	14 (27.5)	24 (25.3)	23 (10.0)	**<0.01**	
**Chronic kidney disease (n, %)**	0 (0.0)	0 (0.0)	0 (0.0)	0 (0.0)	0 (0.0)	6 (6.3)	37 (16.1)	**<0.01**	**<0.01**
**Diabetes mellitus (n, %)**	0 (0.0)	0 (0.0)	0 (0.0)	2 (2.6)	2 (3.9)	16 (16.8)	80 (34.8)	**<0.01**	**<0.01**
**Prematurity** ^b^ **(n, %)**	7 (4.7)	4 (4.3)	3 (6.8)	1 (1.3)	0 (0.0)	0 (0.0)	0 (0.0)	**<0.01**	
**Long term care facility resident**^c^ **(n, %)**	0 (0.0)	0 (0.0)	0 (0.0)	0 (0.0)	0 (0.0)	2 (2.1)	26 (11.3)	**<0.01**	

^a^Immune deficiency includes congenital conditions, HIV/AIDS, solid organ and bone marrow transplants, and patients who received chemotherapy in the six months prior to HRV diagnosis.

^b^Prematurity is defined as birth prior to 37 weeks of pregnancy.

^c^Long term care facility residents were defined as residents of nursing homes or other chronic care facilities.

^d^Trend P was only calculated for binary variables with a monotonic pattern.

Acute bronchiolitis was mainly diagnosed in very young patients (<3 years age), while pneumonia was more likely to be diagnosed in adult patients (>18 years of age) (Cochrane-Armitage trend p < 0.01 for both bronchiolitis and pneumonia). (**Table 2**). The number of asthma exacerbation episodes increased with age among children, but no differences between the age groups were recorded in patients with respiratory failure. Bacterial codetections were recorded in 112 (16.5%) of patients (**Table 3**). Abnormal chest imaging was common in all age groups (18.2% − 36.0%).

**Table 2:** Comparison of clinical presentation in various age groups among HRV hospitalized patients.

	0-1 years	1-3 years	3-5 years	5-18 years	18-40 years	40-65 years	≥65 years	P-value	Trend P
	*n = 148*	*n = 94*	*n = 44*	*n = 76*	*n = 51*	*n = 95*	*n = 230*		
Upper respiratory tract infection (URTI) (n, %)*:	9 (6.1)	5 (5.3)	2 (4.5)	3 (3.9)	8 (15.7)	6 (6.3)	29 (12.6)	**0.03**	
** Tonsillitis (n, %)**	0 (0.0)	0 (0.0)	2 (4.5)	1 (1.3)	0 (0.0)	0 (0.0)	0 (0.0)	**<0.01**	
** Otitis (n, %)**	5 (3.4)	3 (3.2)	0 (0.0)	1 (1.3)	0 (0.0)	0 (0.0)	0 (0.0)	**0.03**	
** Sinusitis (n, %)**	1 (0.7)	0 (0.0)	0 (0.0)	1 (1.3)	0 (0.0)	0 (0.0)	1 (0.4)	0.81	
** Not otherwise specified (NOS) (n, %)**	4 (2.7)	2 (2.1)	0 (0.0)	0 (0.0)	8 (15.7)	6 (6.3)	28 (12.2)	**<0.01**	
Lower respiratory tract infection (LRTI) (n, %)*:	23 (15.5)	19 (20.2)	10 (22.7)	18 (23.7)	20 (39.2)	38 (40.0)	101 (43.9)	**<0.01**	**<0.01**
** Pneumonia (n, %)**	9 (6.1)	6 (6.4)	2 (4.5)	7 (9.2)	12 (23.5)	21 (22.1)	61 (26.5)	**<0.01**	**<0.01**
** Bronchiolitis (n, %)**	14 (9.5)	8 (8.5)	1 (2.3)	0 (0.0)	0 (0.0)	1 (1.1)	3 (1.3)	**<0.01**	**<0.01**
** Chronic obstructive pulmonary disease (COPD) exacerbation (n, %)**	0 (0.0)	0 (0.0)	0 (0.0)	0 (0.0)	0 (0.0)	8 (8.4)	32 (13.9)	**<0.01**	**<0.01**
** Asthma exacerbation (n, %)**	1 (0.7)	8 (8.5)	7 (15.9)	10 (13.2)	7 (13.7)	9 (9.5)	5 (2.2)	**<0.01**	
** Bronchitis (n, %)**	0 (0.0)	0 (0.0)	0 (0.0)	0 (0.0)	2 (3.9)	4 (4.2)	21 (9.1)	**<0.01**	
** Wheezing (n, %)**	1 (0.7)	4 (4.3)	3 (6.8)	3 (3.9)	1 (2.0)	1 (1.1)	0 (0.0)	**0.01**	
** Stridor (n, %)**	4 (2.7)	1 (1.1)	0 (0.0)	0 (0.0)	0 (0.0)	0 (0.0)	0 (0.0)	0.05	
** Respiratory failure (n, %)**	1 (0.7)	1 (1.1)	2 (4.5)	2 (2.6)	0 (0.0)	3 (3.2)	4 (1.7)	0.47	
**Central nervous system (CNS) complications:**	8 (5.4)	7 (7.4)	1 (2.3)	2 (2.6)	6 (11.8)	1 (1.1)	3 (1.3)	**<0.01**	
** Encephalopathy (n, %)**	1 (0.7)	0 (0.0)	0 (0.0)	0 (0.0)	0 (0.0)	0 (0.0)	0 (0.0)	0.68	
** Encephalitis (n, %)**	0 (0.0)	1 (1.1)	1 (2.3)	0 (0.0)	1 (2.0)	1 (1.1)	0 (0.0)	0.26	
** Febrile convulsions (n, %)**	0 (0.0)	3 (3.2)	0 (0.0)	0 (0.0)	0 (0.0)	0 (0.0)	1 (0.4)	**0.02**	
** Non febrile convulsions (n, %)**	7 (4.7)	3 (3.2)	0 (0.0)	2 (2.6)	5 (9.8)	1 (1.1)	2 (0.9)	**0.01**	
**Thromboembolic event (n, %)**	0 (0.0)	0 (0.0)	0 (0.0)	1 (1.3)	1 (2.0)	2 (2.1)	3 (1.3)	0.54	
**Rhabdomyolysis (n, %)**	0 (0.0)	0 (0.0)	0 (0.0)	1 (1.3)	0 (0.0)	1 (1.1)	0 (0.0)	0.34	
**Acute kidney injury (n, %)**	0 (0.0)	1 (1.1)	0 (0.0)	0 (0.0)	0 (0.0)	3 (3.2)	22 (9.6)	**<0.01**	
**Myocardial infarction (n, %)**	0 (0.0)	0 (0.0)	0 (0.0)	0 (0.0)	0 (0.0)	4 (4.2)	5 (2.2)	**0.03**	
**Altered mental status (n, %)**	3 (2.0)	2 (2.1)	2 (4.5)	0 (0.0)	0 (0.0)	2 (2.1)	5 (2.2)	0.64	

*Summation of patients had more than one condition

**Table 3:** Comparison of laboratory, imaging and microbiological findings in various age groups among HRV hospitalized patients.

	0-1 years	1-3 years	3-5 years	5-18 years	18-40 years	40-65 years	≥65 years	P-value	Trend P
	*n = 148*	*n = 94*	*n = 44*	*n = 76*	*n = 51*	*n = 95*	*n = 230*		
**Minimum saturation (mean (SD))**	95.0 [86.8, 96.0]	94.5 [88.2, 96.0]	93.0 [86.0, 96.0]	93.0 [86.0, 96.0]	94.0 [90.0, 96.0]	92.0 [88.0, 94.0]	89.0 [82.0, 93.0]	**<0.01**	
**Maximum creatinine (mean (SD))**	0.3 [0.2, 0.3]	0.3 [0.2, 0.3]	0.3 [0.3, 0.5]	0.6 [0.4, 0.8]	0.8 [0.6, 1.0]	0.9 [0.8, 7.7]	1.4 [0.9, 21.0]	**<0.01**	
**Troponin (mean (SD))**	27.5 [11.4, 462.6]	5.8 [3.5, 10.1]	4.3 [3.3, 5.7]	4.7 [3.5, 38.6]	6.0 [4.2, 21.6]	6.8 [4.4, 34.7]	25.0 [10.3, 75.8]	**<0.01**	
**Maximum C-Reactive Protein (CRP) (mean (SD))**	37.7 (61.1)	63.5 (77.2)	74.1 (77.4)	72.1 (79.9)	97.2 (100.0)	115.8 (104.8)	129.7 (106.8)	**<0.01**	
**Bacterial Codetection (n, %)**	23 (15.5)	16 (17.0)	7 (15.9)	13 (17.1)	4 (7.8)	16 (16.8)	43 (18.7)	0.71	
**Source of codetection:**								0.07	
** Blood (n, %)**	2 (1.4)	3 (3.2)	1 (2.3)	5 (6.6)	1 (2.0)	3 (3.2)	14 (6.1)		
** Urine (n, %)**	7 (4.7)	3 (3.2)	1 (2.3)	2 (2.6)	1 (2.0)	5 (5.3)	19 (8.3)		
** Respiratory (n, %)**	8 (5.4)	3 (3.2)	1 (2.3)	6 (7.9)	1 (2.0)	3 (3.2)	5 (2.2)		
** Other (n, %)**	6 (4.1)	7 (7.4)	4 (9.1)	0 (0.0)	1 (2.0)	5 (5.3)	5 (2.2)		
**Abnormal electrocardiogram (ECG) (n, %)**	16 (10.8)	8 (8.5)	4 (9.1)	4 (5.3)	21 (41.2)	49 (51.6)	149 (64.8)	**<0.01**	**<0.01**
**Abnormal chest imaging**^a^ **(n, %)**	31 (20.9)	24 (25.5)	8 (18.2)	23 (30.3)	13 (25.5)	28 (29.5)	76 (33.0)	0.15	
**Human rhinovirus (HRV) subtype:**								0.5	
** HRV-A (n, %)**	18 (69.2)	3 (50.0)	1 (50.0)	13 (86.7)	6 (85.7)	14 (70.0)	27 (62.8)		
** HRV-B (n, %)**	0 (0.0)	1 (16.7)	0 (0.0)	0 (0.0)	0 (0.0)	0 (0.0)	2 (4.7)		
** HRV-C (n, %)**	8 (30.8)	2 (33.3)	1 (50.0)	2 (13.3)	1 (14.3)	6 (30.0)	14 (32.6)		
**HRV cycle threshold (CT) (median [IQR])**	28.0 [26.0, 30.0]	29.0 [27.2, 30.8]	30.0 [27.8, 31.0]	29.0 [26.0, 30.0]	28.0 [27.0, 30.0]	28.0 [25.5, 30.5]	28.0 [25.0, 30.0]	**0.02**	

^a^Includes chest X-ray, CT, US.

**Fig 1:**
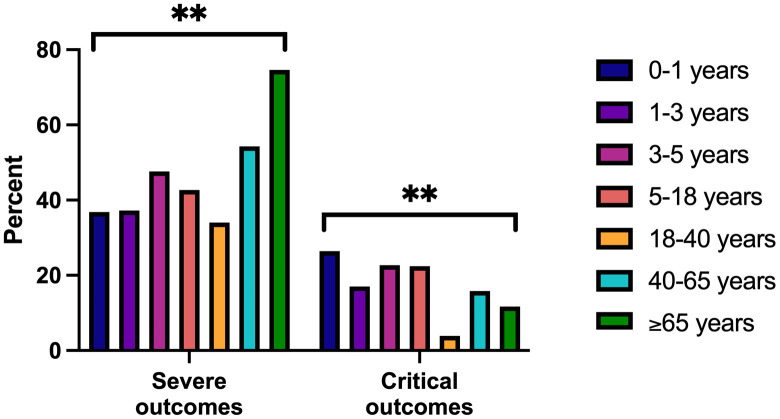
Comparison of severe outcomes as function of saturation, and critical outcomes as function of ICU admission, or mechanical ventilation or amine administration among age groups in hospitalized HRV patients.

The highest percentages of patients with severe outcomes defined by saturation values were recorded among patients in age groups 40–65 years (54.3%) and ≥65 years (74.6%) (**Table 4**, **Fig 1**). The highest percentages of patients with critical outcomes as function of ICU admission or mechanical ventilation or vasopressor support were recorded among patients in age groups 0–1 years (26.4%), 3–5 years (22.7%) and 5–18 years (22.4%). 30-day all-cause mortality was highest in patients aged >65 years (14.3%) (Cochran-Armitage trend p < 0.01). The differences recorded were statistically significant between all age groups (chi-square or Fisher’s exact, p < 0.01). A sensitivity analysis found no differences in the rates of severe and critical outcomes when cases with bacterial co-detection were removed.

**Table 4:** Comparison of outcomes in various age groups among HRV hospitalized patients.

	0-1 years	1-3 years	3-5 years	5-18 years	18-40 years	40-65 years	≥65 years	P-value	Trend P
	*n = 148*	*n = 94*	*n = 44*	*n = 76*	*n = 51*	*n = 95*	*n = 230*		
**Vasopressor support (n, %)**	5 (3.4)	1 (1.1)	0 (0.0)	2 (2.6)	1 (2.0)	10 (10.5)	21 (9.1)	<0.01	
**Mechanical ventilation (n, %)**	3 (2.0)	2 (2.1)	1 (2.3)	3 (3.9)	0 (0.0)	4 (4.2)	7 (3.0)	0.79	
**Intensive care unit (ICU) admission (n, %)**	35 (23.6)	15 (16.0)	10 (22.7)	16 (21.1)	1 (2.0)	5 (5.3)	6 (2.6)	**<0.01**	
**Hospitalization duration (mean (SD))**	4.2 [2.7, 13.1]	4.7 [2.9, 8.9]	4.9 [2.7, 9.7]	7.3 [2.9, 23.4]	2.8 [1.7, 11.5]	2.3 [1.5, 6.3]	3.1 [1.8, 6.3]	**<0.01**	
**30-day all-cause mortality (n, %)**	0 (0.0)	1 (1.1)	0 (0.0)	1 (1.3)	2 (3.9)	6 (6.3)	33 (14.3)	**<0.01**	**<0.01**
**Severe outcomes as function of saturation values ≤92 (n, %)**	50 (36.8)	35 (37.2)	20 (47.6)	32 (42.7)	17 (34.0)	50 (54.3)	170 (74.6)	**<0.01**	
**Critical outcomes as function of ICU admission, or mechanical ventilation or vasopressor support (n, %)**	39 (26.4)	16 (17.0)	10 (22.7)	17 (22.4)	2 (3.9)	15 (15.8)	27 (11.7)	**<0.01**	

### Virologic characteristics of HRV

**Fig 2:**
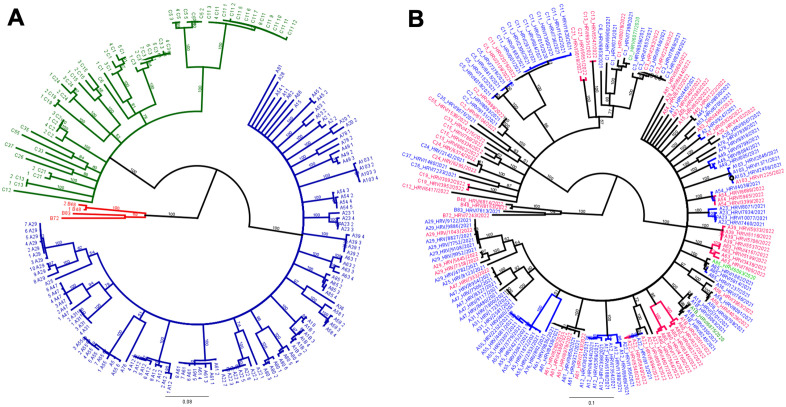
(a) Phylogenetic tree of HRV species (HRV-A, HRV-B and HRV-C) constructed using the Neighbor-Joining method. (b) Phylogenetic tree of HRV species (HRV-A, HRV-B and HRV-C), displaying the study years 2021 (red) and 2022 (blue), constructed using the Neighbor-Joining method.

Virologic characteristics were analyzed from 119 sequenced nasopharyngeal samples with a Ct ≤ 25. Of these samples, 69% (n = 82) were of species A, 2.5% (n = 3) were of species B, and 28.5% (n = 34) were of species C (**Fig 2a**). HRV species A and C were heterogeneous, with 33 and 15 different subtypes detected, respectively. The distribution of species among the various age groups was dominated by HRV-A and HRV-C and did not differ significantly between the age groups (**Table 3**). Phylogenetic analysis revealed distinct clusters of HRV subtypes for each year studied, with the distribution of subtypes varying between the two years analyzed (**Fig 2b**). There was no statistically significant association between Ct and severe or critical outcomes (Spearman’s rank correlation p = 0.21 and 0.50 respectively).

**Fig 3:**
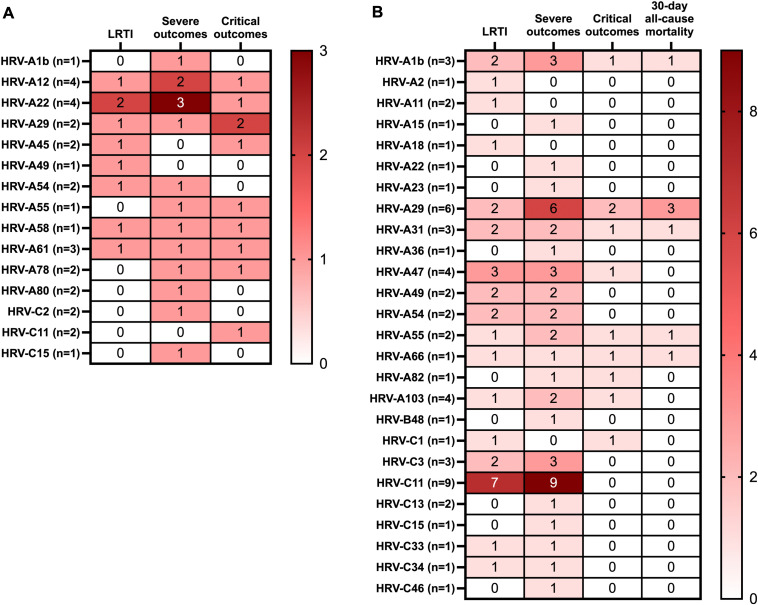
Heat map of HRV serotypes and associated clinical outcomes in hospitalized HRV patients <18 years of age (a) and ≥18 years of age (b). Red represents the highest number, and white represents the lowest number.

Distribution of LRTI, severe and critical outcomes with the various HRV subtypes were analyzed separately in children and adults (**Fig 3a and 3b**). LRTIs in children were reported with subtypes HRV-A 12, 22, 29, 45, 49, 54 and 61, while LRTI in adults were reported with HRV-A subtypes 103, 18, 1B, 2, 29, 31, 47, 49, 54, 55. LRTI associated with HRV-C was found only in adults (subtypes 1, 11, 3, 33 and 34). Severe outcomes in children were reported with HRV-A subtypes 12, A1B, A22, A29, A54, A55, A61, A78, A80 and with HRV-C subtypes C15 and C2. Severe outcomes in adults were reported with HRV-A subtypes 103, 15, 1B, 22, 23, 29, 31, 36, 47, 49, 54, 55, 82 and with HRV-C subtypes 11, 13, 15, 3, 33 and 34. Critical outcomes in children were reported with HRV-A subtypes 12, 22, 29, 45, 55, 58, 61, 78 and with HRV-C subtype 11. Critical outcomes in adults were reported in HRV-A subtypes 1b, 29, 31, 47, 55, 66, 82, 103 and HRV-C 1. 30-day all-cause mortality in adults was reported only with HRV-A (subtypes 1b, 29, 31, 55 and 66). The subtypes with the most reported outcomes were HRV-A22 in children, and HRV-A29 and HRV-C11 in adults.

Critical outcomes for the study population were found to be associated with HRV-A and HRV-C species (Fisher’s exact test p = 0.002 and 0.04, respectively). No statistically significant association was found between HRV-A and HRV-C and severe outcomes (p = 0.512 and 0.334 respectively).

### Comparison of clinical outcomes between patients hospitalized with HRV or hMPV

**Fig 4:**
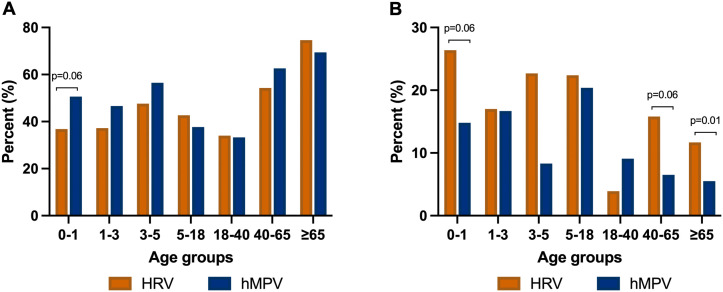
(a) Comparison of severe outcomes as function of saturation among age groups in hospitalized HRV or hMPV patients. (b) Comparison of critical outcomes as function of ICU admission, or mechanical ventilation or amine administration, among age groups in hospitalized HRV or hMPV patients.

A cohort consisting of 855 hMPV patients was compared with the current study cohort (**Fig 4a and 4b**). Of these, 88 (10.3%), 90 (10.5%), 24 (2.8%), 54 (6.3%), 55 (6.4%), 108 (12.6%) and 436 (51.0%) belonged to age groups 0–1, 1–3, 3–5, 5–18, 18–40, 40–65 and >65 years, respectively. There were no statistically significant differences between the various age groups of hospitalized HRV and hMPV patients who experienced severe outcomes. Notwithstanding, the difference between HRV and hMPV patients with severe outcomes in age group 0–1 was borderline statistically significant (p = 0.06). A significantly greater number of HRV patients in the age group >65 years experienced critical outcomes compared with hMPV patients (11.7% versus 5.5%, p = 0.01). A trend for poorer outcomes among HRV infected patients was observed. The differences between HRV and hMPV patients who experienced critical outcomes in age groups 0–1 and 40–65 were borderline statistically significant (p = 0.06).

## Discussion

This retrospective study was conducted in a large cohort of infants, children, adults and the elderly hospitalized with respiratory symptoms between 2020 and 2022 at a tertiary medical center in Israel. Our study analyzed the epidemiological, clinical, and virologic characteristics of HRV infection and evaluated the association between HRV subtypes and clinical outcomes.

HRV was the most frequently detected viral respiratory pathogen during the study period, with the highest number of patients belonging to the youngest and oldest age groups. Acute bronchiolitis was predominantly diagnosed in infants, whereas pneumonia was more frequent in adults across all age group. Rates of asthma exacerbation episodes increased with age among children. No differences between age groups were found among patients with respiratory failure. Severe outcomes (as function of saturation values) were more prevalent in older patients ≥ 65 years, while critical outcomes (ICU admission, mechanical ventilation or vasopressor support) were more common in infants, young children and adolescents. The highest proportion of 30-day all-cause mortality was found in patients aged ≥ 65 years (14.3%).

Previous studies assessing the clinical and epidemiological picture of HRV infection in children and adults have consistently found that bronchiolitis is the leading cause of hospitalization for young patients with HRV infection, and that asthma exacerbation and pneumonia have been associated with respiratory infections with HRV, although the true causal role of HRV may be difficult to establish [1–3,5–7,17–23]. Furthermore, HRV infection may lead to severe or critical outcomes, particularly in extreme life ages [1–3,5,6,21–23].

In order to accurately assess the role of HRV in the etiology of respiratory symptoms observed in this study, we excluded patients with co-detection of other respiratory viruses ((RSV, influenza A & B, parainfluenza, adenovirus, SARS-CoV-2 and hMPV) from the final analysis. To further establish HRV as the primary pathogen, only patients with a high viral load were included, using thresholds of CT ≤ 32 for clinical analyses and CT ≤ 25 for virologic analyses. Several studies have suggested an association between low HRV CT values and increased disease severity, duration or ICU admission; and with increased hospitalization or length of stay [24]. It is important to note, however, that HRV CT values have not been clinically validated, and studies vary in the technique and timing of sample collection. A sensitivity analysis found that removing cases with bacterial co-detection did not alter the rates of severe or critical outcomes, therefore, we did not remove these patients from the final analysis.

Similar studies have attempted to correlate clinical and molecular findings to establish the impact of different HRV species and viral load on disease severity. Mohanty et al [5] examined 52 pediatric cases of acute respiratory infection with HRV, identifying 27 mono-infections, predominantly in children >5 years old. Common symptoms included fever and shortness of breath, with 62.9% of patients presenting with underlying comorbidities, requiring respiratory support. Mono-infections showed lower CT values (<25) compared to co-infections (> 30). Xiao et al [25] evaluated 1,742 nasopharyngeal aspirates from hospitalized children with LRTIs, identifying HRV in 23% of cases, with HRV-A and HRV-C being most prevalent (56% and 25% respectively). While wheezing was more common in HRV-C cases, no differences in disease severity were noted across HRV types. Among children <2 years of age with severe disease, a higher viral load of HRV-A was found in the lower respiratory tract. Ng et al [26] investigated HRV in 3,935 patients, sequencing 976 positive cases (49% were HRV-A, 38% were HRV-C, and 13% were HRV-B), observing higher viral loads and more severe symptoms with HRV-C. Viral loads declined significantly after symptom onset.

From the 119 sequenced nasopharyngeal samples with a high viral load (CT ≤ 25), 69%, 2.5% and 28.5% were represented by species HRV-A, HRV-B, and HRV-C. Various studies have evaluated the differences in clinical symptoms and outcomes according to HRV species. HRV-C species have been identified as a risk factors for wheezing and more severe forms of HRV infections in pediatric populations and, less frequently, in adult populations [3,17–19,21–23,27–29]. However, other studies reported no clear link between HRV species and clinical outcomes [24,26,30]. In our study, HRV-A and HRV-C samples exhibited considerable genetic subtype diversity, which is consistent with previous studies on community wide and hospitalized cohorts, which describe extensive genotype variability in HRV-infected individuals [31–36]. LRTI associated with HRV-C was found only in adults while severe and critical outcomes with HRV-A and HRV-C were reported in both children and adults. 30-day all-cause mortality in adults was reported only with HRV-A.

Our findings suggest that there may be an association between HRV subtype and clinical outcome, and that different subtypes were linked to different clinical outcomes in children and adults. The subtypes with the highest number of reported outcomes (LRTI, severe outcomes, critical outcomes and 30-day all-cause mortality) were HRV-A22 in children, and HRV-A29 and HRV-C11 in adults. Limited data has been reported on the association between specific HRV subtypes and clinical outcomes. Liu et al [37] reported a severe case of HRV-A45 in a previously healthy, immunocompetent 10-year-old who developed central nervous system involvement and viral sepsis, while Yan et al [38] identified HRV-B91 in a 60-year-old woman with severe community-acquired pneumonia. Giardina *et al* [39] described two nosocomial outbreaks within 30-day periods in a NICU in Italy, with 4 patients infected by HRV-C43, and subsequently 4 patients infected by HRV-A89. Bruning *et al* [13] compared HRV infections in 49 hospitalized and 65 non-hospitalized children and found that subtypes HRV-A12, HRV-B6, HRV-B102 and HRV-C2 were unique to hospitalized children. Naeem *et al* [40] found that the predominant subtypes among 102 HRV-infected pneumonia patients aged <15 years were HRV-A101 and HRV-C8.

When HRV patients were compared with patients diagnosed with hMPV, no differences were found between the various age groups of hospitalized HRV and hMPV patients who experienced severe outcomes, but more HRV patients aged ≥65 years experienced critical outcomes compared with hMPV patients. Garcia-Garcia *et al* [41] compared hMPV infections in hospitalized children with other respiratory viruses and found no differences in ICU admission between hMPV and HRV patients (2.2% and 2.1%, p = 0.477), however a greater proportion of hMPV patients had lower saturation levels (<95%) compared with HRV patients (62.4% versus 51%, p = 0.021). Asner et al [42] found that HRV-infected children experienced a more severe clinical course than those infected with RSV and influenza, though the HRV-infected children often had significant comorbidities.

Our study was retrospective, and therefore a major limitation of this study derives from the data collection process, based on computerized patient medical records. Some information regarding the patient’s detailed medical history may not have been recorded, particularly if the patients had additional visits at other medical centers or private clinics. Being a tertiary referral hospital could also have biased our findings towards more serious illness, as is reflected by the characteristics of the included patients (of them a considerable amount suffered from chronic underlying illness and had severe or critical outcomes). In addition, we could not relate with certitude the day of HRV sampling to the onset of symptoms in all patients and, of course, could not exclude the possibility of asymptomatic HRV carriage in some patients. Furthermore, the number of respiratory samples submitted for sequencing and genomic analysis was not large enough to allow an appropriate analysis of disease severity as function of HRV types.

In conclusion, this study reported on the extensive involvement of genetically diverse HRV in acute respiratory disease in a large, hospitalized patient population presenting with signs and symptoms of an acute respiratory infection, and suggests that HRV infection may be associated with considerable morbidity rates in both children and adults.

## Supporting information

S1 File Supporting information table: Primers used for HRV species A, B and C sequencing. (DOCX)
