# Supplementary material for: Clinical characteristics, outcomes, and subtype diversity in hospitalized human rhinovirus (HRV) patients
Source: PLoS One. 2025 Nov 5;20(11):e0335739. doi: 10.1371/journal.pone.0335739 (PMC12588530; doi:10.1371/journal.pone.0335739)
Supplement: S1 File — (DOCX) [file pone.0335739.s001.docx]

**Supporting Information Table: Primers used for HRV species A, B and C sequencing [17].**

| **Sequence 5' 3'** | **Orientation** | **Target** | **Position** | **Product size** |
| --- | --- | --- | --- | --- |
| CCGGCCCCTGAATGYGGCTAA | Outer sense | VP4/VP2 | 458 | 667 |
| ACATRTTYTSNCCAAANAYDCCCAT | Outer inner sense |  | 1,125 |  |
| ACCRACTACTTTGGGTGTCCGTG | Inner sense |  | 547 | 540 |
| TCWGGHARYTTCCAMCACCANCC | Inner antisense |  | 1,087 |  |
| CAAGCACTTCTGTYWCCCC | Sense | 5' UTR | 179 | 372 |
| ACGGACACCCAAAGTAG | Antisense |  | 551 |  |

**Reference:**

[17] Jans DA, Ghildyal R. Rhinoviruses Methods and Protocols Methods in Molecular Biology 1221 [Internet]. Available from: http://www.springer.com/series/7651
